# Coaching knowledge, sport emotion, and perceived performance in Korean judoka

**DOI:** 10.3389/fpsyg.2025.1615383

**Published:** 2025-09-02

**Authors:** KwangWoo Nam, JungHoon Ha, SangJin Yoon

**Affiliations:** ^1^Department of Physical Education, Republic of Korea Naval Academy, Changwon, Republic of Korea; ^2^Department of Physical Education, Korea National Sport University, Seoul, Republic of Korea; ^3^Sport Coaching Science, Graduate School of Health and Sport Science, Nippon Sport Science University, Setagaya-ku, Japan

**Keywords:** Korean, judo, coaching knowledge, sport emotion, perceived performance

## Abstract

**Introduction:**

Perceived performance is associated with coaches’ expert knowledge and athletes’ sport emotion; however, the relationships among these variables have not been studied. This study aimed to investigate the structural relationships between the coaching knowledge of judo coaches and the sport emotion and perceived performance of Korean judo practitioners, or “judoka.”

**Methods:**

Data from *n* = 249 Korean judoka were collected via questionnaire and analyzed using frequency analysis, confirmatory factor analysis, reliability analysis, correlation analysis, and structural equation modeling.

**Results:**

Coaching knowledge significantly increased positive emotion (*β* = 0.554, *p* < 0.001) and perceived performance (*β* = 0.333, *p* < 0.001), and significantly decreased negative emotion (*β* = −0.356, *p* < 0.001). Positive emotion significantly increased perceived performance (*β* = 0.638, *p* < 0.001), whereas negative emotion did not decrease perceived performance (*β* = −0.029, *p* = 0.427).

**Conclusion:**

High-quality coaching knowledge is associated with higher levels of athletes’ positive emotion, reduced levels of negative emotion, and enhanced perceived performance. To optimize perceived performance, coaches should further develop their coaching expertise, while athletes should actively engage in emotion regulation strategies.

## 1 Introduction

Judo is a sport in which two athletes of similar stature hold, throw, pin, squeeze, and twist each other. Judo has received much attention since being introduced at the Tokyo Olympics in 1964 (Nam et al., 2024) and is a martial arts discipline with complex physical demands, including adequate anaerobic and aerobic fitness, strength, and power. Even among athletes of the same weight class, variations in stamina, strength, and technique are observed, prompting intensive and multidimensional training for performance improvement (Kim and Nam, 2020). In particular, Korean judoka are recognized for their highly systematic training programs, which have contributed to South Korea’s consistent success on the world stage, ranking third globally behind Japan and France.

Thus, we aimed to explore the factors involved in the elevation of Korean judoka’s perceived performance to a world-class level. Specifically, we used perceived performance as the dependent variable, focused on coaching knowledge as a closely related predictive variable in terms of the coach’s role, and focused on sport emotion for psychological factors during training and matches.

While our empirical sample comprises Korean judo coaches and athletes, the underlying mechanisms—systematic training design, emotional support, and strategic feedback—are fundamental to coaching across many sports and cultures (Choi et al., 2020; Nam et al., 2024). Indeed, while Korean judo’s distinctive integration of collective discipline, hierarchical coach–athlete relationships, and highly structured technical drills may intensify these effects, the core relationships uncovered here likely extend to other sports coaches and national contexts. Future research should validate these pathways in team, individual, and combat sports beyond Korea to confirm their broader applicability.

Nevertheless, judo also differs in fundamental ways from many non-combat sports such as football, tennis, or swimming. In football, decision-making and performance are distributed among multiple players, allowing shared responsibilities and compensations during play (Andrzejewski et al., 2016; Pan et al., 2023). In tennis, individual athletes frequently experience rapid emotional fluctuations due to the back-and-forth nature of rallies and scoring (Bois et al., 2009; Stein et al., 1995). Swimming emphasizes rhythm and consistency under high physiological strain, with limited room for strategic interaction during competition (De Jesus et al., 2016; Yin et al., 2023). In contrast, judo is a combat sport involving continuous physical interaction, intense psychological pressure, and real-time interpretation of an opponent’s body cues and balance. These unique demands foster a performance environment where emotional control, tactical anticipation, and close physical confrontation are central to success (Coswig et al., 2018; Franchini et al., 2013). Therefore, findings from judo—especially those involving the regulation of sport emotion—may not be directly generalizable to all other athletic domains.

Korean judo coaches, in particular, differ from many of their international counterparts in several cultural and methodological aspects. Coaching in Korea often reflects Confucian hierarchical values, resulting in a top-down coaching structure where the coach’s authority is rarely questioned. This differs from more athlete-centered approaches common in Western countries that emphasize individual autonomy and dialogue (Sakalidis et al., 2023; Kim and Tak, 2025; Nam et al., 2023). Korean coaches also place a strong emphasis on repetitive, highly disciplined training routines, which are designed to cultivate not only technical mastery but also psychological resilience (Nam and Cho, 2014; Nam et al., 2024).

These unique features of Korean judo coaching make this population especially relevant for examining the interaction of coaching knowledge, sport emotions, and perceived performance. These distinctive characteristics may stem from Korea’s collectivist cultural values, which emphasize emotional stability and group harmony over individual expression. Prior research in East Asian sports psychology has noted that athletes from collectivist societies often develop strong emotional regulation strategies to maintain performance and team cohesion (Kim and Nam, 2020).

For the purpose of this study, we clearly distinguish between ‘athletic performance’ and ‘perceived performance.’ ‘Athletic performance’ refers to the objectively measurable outcomes and skills demonstrated by the athletes (Ginevičienė et al., 2022; Guth and Roth, 2013), whereas ‘perceived performance’ is defined as the athletes’ subjective evaluation of their own performance (Lee, 2000; Mamassis and Doganis, 2004). We hypothesize that coaching knowledge exerts an indirect effect on perceived performance through its positive influence on sport emotion.

To comprehensively evaluate athletes’ performance, this study incorporates psychological variables—specifically sport emotion—into the main model because these variables capture the subjective evaluations and internal states that objective measures of athletic performance cannot fully reflect. This approach is grounded in theoretical frameworks suggesting that an athlete’s self-assessment (perceived performance) is mediated by emotional and cognitive processes, which are considered to be associated with motivation, confidence, and overall performance appraisal (Fransen et al., 2018; Hanin, 2000; Jackson et al., 2001; Moen et al., 2019). Within this conceptual framework, coaching knowledge is hypothesized to indirectly influence perceived performance by modulating athletes’ emotional responses during training and competition, thereby enhancing their subjective assessment of success (Hanin, 2000; Mccarthy, 2011). This emphasis on perceived performance recognizes the critical role that emotional factors play in how athletes interpret and evaluate their own performance. These emotional components help to describe how athletes experience their own success and challenges, thereby offering a more nuanced understanding of performance beyond mere physical metrics (Fransen et al., 2018; Gellatly and Meyer, 1992; Moen et al., 2019).

The majority of studies on coaching knowledge cited in this manuscript have been conducted by researchers in Korea or East Asia. These studies primarily investigate athletes and coaching practices within the regional context, which inherently reflects the psychological characteristics and cultural nuances of East Asian, particularly Korean, athletes. This deliberate focus frames our research within the unique cultural and psychological context of Korean judoka. Considering Korea’s highly competitive judo environment, this study builds upon these regional findings by examining the structural relationships among coaching knowledge, sport emotion, and perceived performance, specifically among Korean judoka. Hence, our research aims to extend the current understanding of how coaching practices influence perceived performance in a setting characterized by unique training cultures and competitive demands.

In sports, coaches are crucial for improving the performance of teams and athletes. They fulfill diverse roles, from establishing thorough plans for systematic training to enhancing individual athletes’ stamina and technique, providing strategies and tactics for effective performance and managing athletes’ condition (Nam and Cho, 2014). Coaching function, as the comprehensive term for all coaching activities, can be considered the overall product of an organic combination of various heterogeneous functions. Coaching function, with its complex mechanisms, is not simply an arbitrary, situational, or improvisational act, but is expressed according to fundamental, consistent behavioral principles, referred to as “coaching knowledge” (Chung and Huh, 2006). In other words, the coaching knowledge of judo coaches refers to the systematization of several judo-related functions that have accumulated with long-term experience and learning as an athlete and/or a coach, as well as the teaching ability to efficiently and effectively convey this knowledge to athletes (Nam and Cho, 2014). Chung and Huh (2006) categorized coaching knowledge into five factors: (1) knowledge of training methods for efficient training, (2) knowledge of coaching theory, including discipline-specific characteristics and information, (3) knowledge of training programs for systematic training, (4) understanding of athletes, and (5) knowledge of strategy for match situations. Previous studies on coaching knowledge (Choi and Ha, 2020; Kim and Choi, 2017; Nam and Cho, 2014; Yoo et al., 2018) found that it had positive effects on athletes’ exercise immersion, behaviors, and motivation, as well as athletic performance and trust in coaches.

The successful performance of athletes in competition is influenced by various factors, including physical, mechanical, physiological, and psychological factors. In particular, the importance of psychological factors is emphasized when athletes’ skill and physical ability are well-matched, and sport emotion has received much attention as a factor affecting athletic performance (Hanin, 2004; Kim et al., 2023). Sport emotion refers to the emotions generated during physical activity. An athlete’s emotional state is crucial for their everyday mental health, as well as for maintaining optimal performance during training and competition (Lee and Yoo, 2023). Sport emotion includes both positive feelings such as pleasure and joy, and negative ones such as anxiety and disappointment, based on the athlete’s psychological evaluation of competitive tension (Lazarus, 2006; Watson and Clark, 1994). Positive emotions experienced through sports include pleasure, joy, and happiness, while negative emotions include sadness, disappointment, anxiety, and depression (Rejeski et al., 1992). Athletes inevitably experience positive emotions regarding their goals for victory and negative emotions regarding defeat. Hence, athletes must focus on their emotions and deal with them appropriately (Park and Jeong, 2022). For judoka, it is essential to cope appropriately with sport emotion during training and competition as it is closely related to athletic performance.

Athletic performance is the totality of skills and abilities demonstrated by an athlete during sports. It is the ability of an athlete that ultimately drives sporting events and is rewarded with “victory” (Jang and Kong, 2022). Athletic performance is not elucidated by single medals or records but refers to the sporting ability of an individual learned over long periods of systematic training and effort, which includes the ability to produce positive results in competition (Kim and Bang, 2022). Particularly, judo is a discipline with weight classes, where two athletes of similar stature compete for victory. This means that exceptional athletic performance is influenced not only by physical ability and skill but also by tactics, strategies, and psychological factors. Additionally, during matches, deep focus, attention, and rapid judgment are essential (Song, 2021). Athletic performance is affected by coaches’ expert knowledge and athletes’ sport emotion, which are the two variables we aimed to analyze in this study (Cha and Jo, 2017; Chung, 2017; Yoo et al., 2018).

Research investigating the relationship between coaching knowledge and sport emotion to confirm a direct relationship between these two variables is scant. However, studies have confirmed that athletes’ sport emotion is positively affected by several variables related to the coach’s role, including self-perception, self-management, social perception, emotional leadership in managing relationships, charisma, individual consideration, intellectually stimulating transformational leadership, democratic behavior, leadership types involving positive reinforcement, experience building, social/psychological function, mentoring, and autonomy support (Hong et al., 2013; Hong and Choi, 2022; Kim, 2012; Kim, 2011; Kim and Choi, 2014). Based on these findings, we can predict that coaching knowledge will have beneficial effects on sport emotion. Previous studies have reported that both coaching knowledge and sport emotion have positive effects on athletic performance (Cha and Jo, 2017; Chung, 2017; Lee et al., 2019; Yoo et al., 2018), which suggests that the coaching knowledge of judo coaches and the sport emotion of judoka could improve perceived performance.

Based on these previous studies, we aimed to investigate the structural relationships between the coaching knowledge of judo coaches and the sport emotion and perceived performance of judoka. This study is significant because the relationships between these variables have not been studied. Moreover, we elucidate the essential functions of coaches in developing the athletic performance of Korean judoka, who have demonstrated excellent performance at an international level. We anticipate that our findings could be used as basic and educational data to inform the roles of coaches and related parties and the efforts required from them to ensure the advancement of athletic performance in judo and other similar disciplines.

In summary, while prior research has identified positive links among coaching knowledge, sport emotion, and athletic performance, few studies have explored how these variables interact to influence perceived performance—particularly in the context of Korean judo. Unlike prior studies such as Horn et al. (2001) and Yang and Nam (2019), which primarily examined how coaching-related and situational factors affect objective athletic performance, this study uniquely investigates how coaching knowledge and sport emotion influence athletes’ subjective evaluation of their performance—that is, perceived performance. This distinction is critical because perceived performance is shaped not only by observable outcomes but also by internal psychological states, providing insight into athletes’ self-assessment and emotional experiences that traditional performance metrics often overlook. Accordingly, this study fills an important research gap by investigating the structural relationships among coaching knowledge, sport emotion, and perceived performance, offering a novel perspective on how coaching influences athletes’ subjective experiences in emotionally demanding sports like judo. Furthermore, the insights generated in this study are not limited to Korean judo coaches; they may also apply to sports coaches in general or coaches in other countries, given the universal importance of coaching expertise and emotional processes in athlete self-evaluation.

## 2 Conceptual framework

### 2.1 Relationship between coaching knowledge and sport emotion

Outstanding coaches fulfill many roles based on their own experience and learning, such as thoroughly analyzing information and data, which is further employed to select and utilize athletes and construct tactics and strategies, as well as implementing systematic training methods, managing athletes, and working in harmony with athletes during live competitions. In other words, coaches accumulate knowledge through extensive trial-and-error and learning from former experience as an athlete and experience as a coach. This coaching-related knowledge is referred to as “coaching knowledge.” Thus, coaching knowledge is a consistent behavioral principle in which a diverse combination of functions is organically expressed and includes knowledge of coaching theory, training methods, training programs, match strategy, and understanding required to instruct athletes (Chung and Huh, 2006). Coaches are not born with coaching knowledge; it is acquired through experience and learning (Ericsson and Smith, 1991). Excellent coaches have to invest significant effort to acquire a high level of coaching knowledge. Nam et al. (2023) reported that coaches not only acquire coaching knowledge through their own experience as athletes but also through engaging in significant efforts as a coach, such as designing training methods and programs, choosing what theoretical background to use as the basis for strategy during matches, and understanding the athletes who implement these strategies.

Moreover, they reported that these efforts have positive effects on exercise passion, a motivational force that helps athletes achieve their personal objectives. Nam and Cho (2014) reported that to efficiently coach judoka, coaches need to make persistent efforts to acquire systematized expert knowledge of the discipline; this knowledge is not native but rather acquired through learning and ample experience. Moreover, they reported that this knowledge can be used to increase athletes’ exercise immersion, which is a form of intrinsic motivation that drives athletes to actively participate in matches and training and is foundational to improving athletic performance.

Park and Kim (2016) illustrated that coaching knowledge includes training methods focused on improving athletes’ performance through effective training, bold match strategies based on thorough analysis, systematic and scientific training programs, the maintenance of warm interpersonal relationships in both training and match situations, and consideration for athletes. According to their study, better coaching knowledge could improve athletes’ self-management of physical condition, personal relationships, mentality, and training, as well as sports confidence, including perceived performance, physical preparation, and social support.

Although some individual paths (e.g., coaching knowledge and athletic performance) have been tested in prior studies, the overall model tested here—linking coaching knowledge, sport emotion, and perceived performance—has not previously been examined. Thus, each hypothesis includes at least a partial extension or novel contribution to the existing literature.

**Figure 1:**
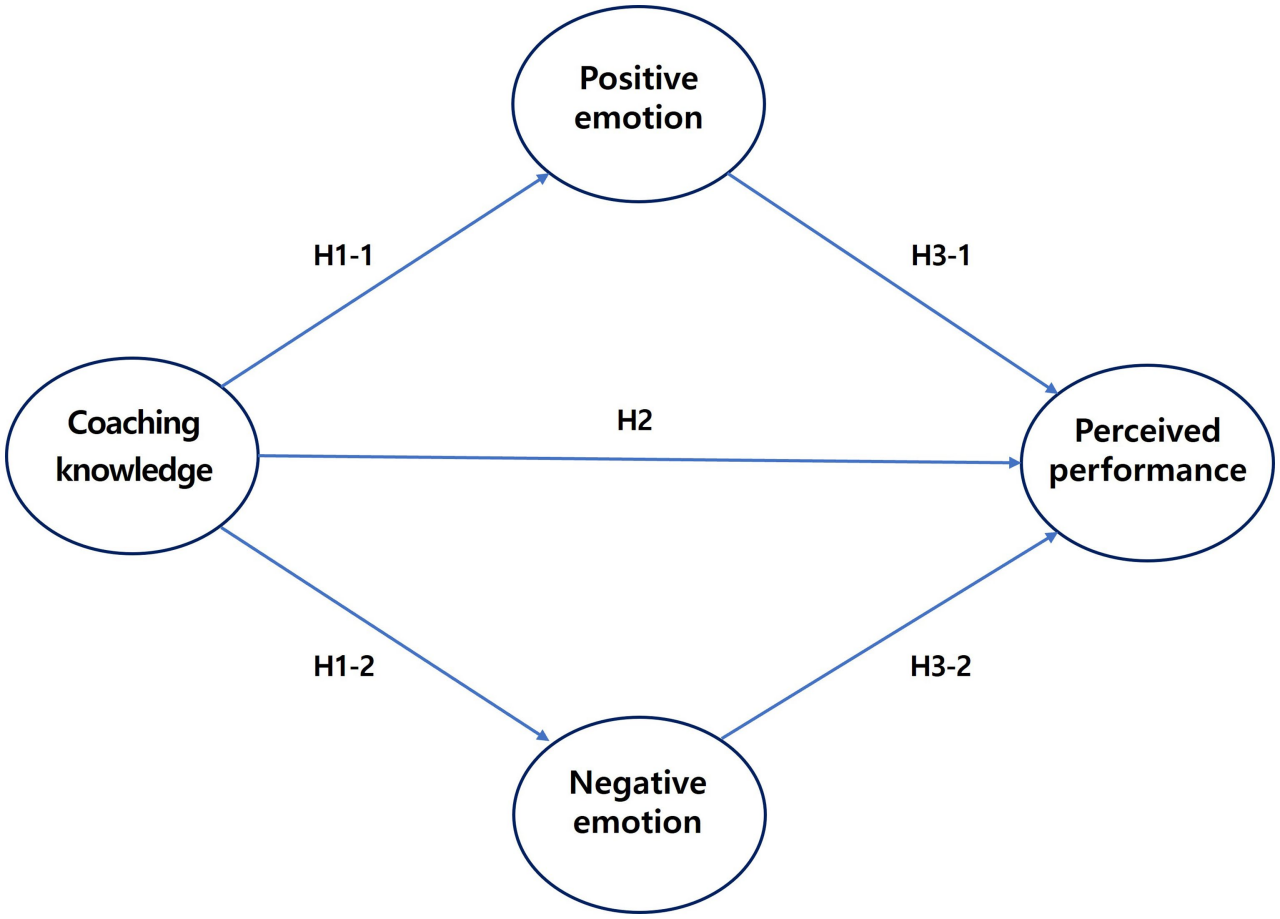
Research model and hypotheses.

Based on these prior studies, we established the following hypotheses for this study (Figure 1):

*H-1.* The coaching knowledge of judo coaches influences the sport emotion of athletes.

*H-1-1.* The coaching knowledge of judo coaches increases the positive emotion of athletes.

*H-1-2.* The coaching knowledge of judo coaches decreases the negative emotion of athletes.

### 2.2 Relationship between coaching knowledge and perceived performance

Athletic performance is the totality of an athlete’s functional and physical ability expressed in sporting situations (Cho, 2023), also referred to as “competitive performance.” While discussing athletic performance, Nam et al. (2024) reported that the role of coaches is essential and that they perform various roles, as mentioned previously. In other words, coaches attempt to integrate their knowledge into training to improve athletes’ performance. To provide athletes with effective training and help them achieve successful outcomes in competition, coaches need to have expert coaching knowledge, including knowledge of training, strategies and tactics for different match situations, and analysis of individual and team skills (Chung, 2004; Chung and Huh, 2006). Among studies analyzing the relationship between coaches’ coaching knowledge and athletes’ performance, Cha and Jo (2017) reported more positive changes regarding the coach’s training programs, match strategy, athlete understanding, coaching theory, and training methods when athletes perceived a coach to have a high level of coaching knowledge, and this change ultimately had direct effects on the athletes’ performance. Nam et al. (2023) reported that, in addition to expert knowledge on each discipline, coaches need to make a devoted effort to acquire methodological and strategic knowledge to provide athletes with proper instruction in training and match situations, and this effort not only improved athletes’ performance but also had a major effect on their future lives. Based on these prior studies, we established the following hypothesis for this study:

*H-2.* The coaching knowledge of judo coaches increases athletes’ perceived performance.

### 2.3 Relationship between sport emotion and perceived performance

Sport emotion is a concept that encompasses all interest and pleasure that reduce negative factors and positively influence athletes’ emotional experiences during exercise and sports (Hwang, 2018). In terms of exercise participation, athletes’ sport emotion greatly impacts sport performance (Lazarus, 2000). Sport emotion can be broadly divided into positive and negative emotions. Negative emotion negatively influences exercise performance and exercise behaviors, and can form a basis for avoiding exercise participation (Park and Kang, 2023). Emotional changes in athletes significantly influence training and matches; in particular, negative emotion not only causes confusion in athletes’ performance but can also act as a psychological burden (Lemyre et al., 2006). Conversely, positive emotion has positive effects on sports performance and athletic performance (Chung, 2017; Kim et al., 2011). Moreover, positive sport emotion has important effects on team performance, team cohesion, athlete satisfaction, and exercise outcomes (Choi and Yoo, 2011; Crombie et al., 2009; Kim and Lee, 2012). These findings demonstrate that sport emotion has positive effects on athletic performance. Hence, the following hypotheses were formulated for this study:

*H-3.* The sport emotion of judoka influences their perceived performance.

*H-3-1.* Positive sport emotion of judoka increases their perceived performance.

*H-3-2.* Negative sport emotion of judoka decreases their perceived performance.

## 3 Materials and methods

### 3.1 Participants

The study population was judoka officially registered on school, university, or business judo teams affiliated with the Korean Sport and Olympic Committee in 2023, and participants were selected using convenience sampling. Specifically, the investigators, who have a background as judoka and judo coaches, reached out to coaches they already knew and administered a questionnaire to athletes on the school and business teams where these coaches worked. To mitigate potential response biases arising from demand characteristics or social pressure (e.g., reluctance to express negative perceptions of coaching), all participants were assured of complete anonymity and confidentiality. Additionally, the questionnaire instructions explicitly stated that individual responses would not be disclosed to their coaches, and all questionnaires were distributed and collected by independent research staff, with coaches absent during completion and responses were collected without coaches present to ensure independence. All questionnaires were administered concurrently within 24 h after a competition, ensuring that the responses for coaching knowledge, sport emotion, and perceived performance were captured under similar conditions. Before the questionnaire, the objectives and intentions of the study were thoroughly explained to the participants, and consent to participate in the questionnaire was obtained.

Due to logistical constraints, the questionnaire was administered at a single time point: within 24 h after the completion of a competition. Specifically, the questionnaires were administered after post-match recovery sessions to ensure that responses were obtained under physically and mentally stable conditions. This approach minimized potential fatigue-related biases that could influence athletes’ perceptions of coaching and performance. Furthermore, measures such as ensuring anonymity and emphasizing the importance of honest responses were implemented to mitigate potential response biases, especially among athletes with less than 5 years of experience. This study was approved by the Korea Public Institute Review Board (protocol code P01-202502-01-026) and conducted in strict accordance with ethical guidelines to protect participants’ rights and confidentiality.

After excluding nine responses judged to be unreliable—either because all questions had been answered with the same number or due to missing answers—data from 249 participants were included in the final analysis. Table 1 presents the general characteristics of the participants, including their team levels, which generally correspond to the following age groups: middle school (13–15 years), high school (16–18 years), university (19–22 years), and business team (23–30 years). Based on this classification, the estimated mean age of the sample is approximately 17.45 years.

**Table 1:** Characteristics of the participants (*n* = 249).

Variable	Division	n	%
Gender	Men	188	75.5
	Women	61	24.5
Team level	Middle school (13–15 years)	83	33.3
	High school (16–18 years)	84	33.7
	University (19–22 years)	68	27.3
	Business team (23–30 years)	14	5.6
	Mean age (years)	249	17.45
Exercise experience	< 5 years	127	51.0
	< 10 years	85	34.1
	< 15 years	27	10.8
	> 15 years	10	4.0
Medals awarded	International competition	19	7.6
	National competition	116	46.6
	City/province-wide competition	52	20.9
	No medal placements	62	24.9

n, number of participants.

All statistical analyses were conducted using SPSS v21 and AMOS v21.

### 3.2 Questionnaire instruments

We used questionnaires and scales that have been validated in previous studies for the questionnaire instruments in this study. The questionnaire items were edited and supplemented to fit the objective and intentions of our study. The content validity was tested by consensus among a panel of experts, consisting of four doctors in a field related to Sports Science. These experts had extensive research and practical experience in sports coaching, psychology, and performance analysis, making them well-qualified to evaluate the appropriateness and comprehensiveness of the questionnaire items. The panel assessed the validity of the items based on their relevance, clarity, and alignment with established theoretical frameworks in sports science.

First, to measure coaching knowledge, we used a questionnaire instrument developed by Chung and Huh (2006) to analyze coaching knowledge among coaches in sports departments that had been tested for structural validity. The Coaching Knowledge Scale consists of five subfactors and 25 items in total, with five items on training methods, coaching theory, training programs, athlete understanding, and match strategy, respectively (training methods; “Uses training time effectively,” *α* = 0.938; coaching theory; “Accurately conveys exercise skills,” *α* = 0.900; training programs; “Implements training programs logically,” *α* = 0.929; athlete understanding; “Has a sound understanding of the personality and characteristics of each individual athlete,” *α* = 0.894; match strategy; “Does not miss the moments during matches when a change of strategy is needed,” *α* = 0.921). Second, to measure sport emotion, we used a scale based on the Manual for the Positive and Negative Affect Schedule by Watson and Clark (1994), which was validated and used by Ha and Hong (2022) to measure sport emotion in South Korean athletes. The Sport Emotion Scale consisted of two subfactors and ten items in total, with five items each on positive emotion and negative emotion, respectively (positive emotion; “I feel happy during exercise,” *α* = 0.936; negative emotion; “I feel anxious during exercise,” *α* = 0.910). Third, to measure perceived performance, we used a scale developed by Lee (2000) to more finely classify perceived performance by combining the Perceived Performance Scale developed by Mamassis and Doganis (2004) with the Task and Ego Orientation in Sport Questionnaire (TEOSQ) developed by Duda (1989). The Perceived Performance Scale consisted of five items on performance success, five items on performance maturation, and four items on psychological maturation (performance success; “I feel successful when I use a new technique with good results,” *α* = 0.888; performance maturation; “I am able to solve judo-related problems myself,” *α* = 0.871; psychological maturation; “I think that exercise, in itself, is an enjoyable activity,” *α* = 0.876).

Except for the questions about general characteristics, the items in the aforementioned instruments were scored on a 5-point Likert scale ranging from 1 (“*strongly disagree*”) to 5 (“*strongly agree*”). The specific content of the items is depicted in Table 2.

**Table 2:** Composition of the questionnaire items.

Category	Constituent factors (item count)	Total item count
General characteristics	Gender (1)	4
	Team level (1)	
	Exercise experience (1)	
	Competition record (1)	
Coaching knowledge	Training methods (5)	25
	Coaching theory (5)	
	Training programs (5)	
	Athlete understanding (5)	
	Match strategy (5)	
Sport emotion	Positive emotions (5)	10
	Negative emotions (5)	
Perceived Performance	Performance success (5)	14
	Performance maturation (5)	
	Psychological maturation (4)	
Total		53

### 3.3 Data analysis

In this study, data analysis was performed in the following order. First, frequency analysis was conducted to examine the participants’ general characteristics. Next, confirmatory factor analysis and reliability analysis were conducted to verify the construct validity, convergent validity, and internal consistency of the questionnaire instruments (these analyses are reported in the Results section). Then, correlation analysis was performed to assess the relationships between subfactors. Finally, structural equation modeling was used to test the hypotheses.

We performed statistical analyses to test the research hypotheses, including structural equation modeling (SEM), which allows for the simultaneous testing of multiple latent variable relationships and accounts for measurement error (Stephenson et al., 2006; Williams et al., 2009).

## 4 Results

### 4.1 Reliability and validity of the research instrument

We followed the listed procedure to ensure the validity and reliability of the questionnaire instruments. First, we used confirmatory factor analysis (CFA) to ensure the construct validity of the instruments. Specifically, in the initial CFA, we included all items from the Coaching Knowledge Scale (5 factors × 5 items = 25 items), Sport Emotion Scale (2 factors × 5 items = 10 items), and Perceived Performance Scale (3 factors × 5, 5, and 4 items = 14 items), totaling 49 items across 10 latent variables. We observed *χ*^2^(*df*) = 1854.967 (1082)/*p* < 0.001, SRMR = 0.046, TLI = 0.929, CFI = 0.927, and RMSEA = 0.050, indicating that the fit indices were good. However, Question 5 of the Perceived Performance Scale demonstrated an SMC value of 0.159, indicating that the ability to explain variance in the measured variable was lacking (Song, 2016). Therefore, we removed Question 5 for perceived performance, repeated the confirmatory factor analysis, and, as exhibited in Table 3, observed good fit indices, with *χ*^2^(*df*) = 1728.327(1035)/*p* < 0.001, SRMR = 0.045, TLI = 0.928, CFI = 0.934, and RMSEA = 0.052, demonstrating the construct validity of the instruments. Second, we confirmed the construct validity of the questionnaire instruments based on convergent validity (AVE) and construct reliability (CR). Previous studies (Bae, 2017; Hair et al., 2006) have advised checking for AVE (≥0.5) and CR (≥0.7) values based on the confirmatory factor analysis results. The calculated AVE was 0.624–0.792 and CR was 0.891–0.950, demonstrating construct validity. Third, we verified the internal consistency of the questionnaire instrument by performing a reliability analysis using Cronbach’s *α*. When we performed the reliability analysis, we observed high Cronbach’s *α* values of 0.894–0.938 for coaching knowledge, 0.910–0.936 for sport emotion, and 0.871–0.888 for perceived performance, demonstrating internal consistency.

**Table 3:** Validity and reliability analysis.

Variables		Item	*β*	S. E.	C. R. (*t*)	*p*	AVE	CR	*α*
Coaching knowledge	Training methods	Appropriately adjusts the time and quantity of training	0.880				0.792	0.950	0.938
		Effectively adjusts the quantity of training while coaching	0.912	0.048	21.567	0.001			
		Uses training time effectively	0.889	0.046	20.356	0.001			
		Appropriately splits training time when coaching	0.890	0.048	20.391	0.001			
		Changes training contents and order to prevent training from becoming boring	0.783	0.061	15.895	0.001			
	Coaching theory	Effectively explains the importance of each skill during training	0.802				0.708	0.924	0.900
		Is highly competent at teaching judo	0.846	0.066	15.455	0.001			
		Encourages questions to check whether the athlete has understood accurately	0.764	0.072	13.451	0.001			
		Accurately conveys exercise skills	0.870	0.066	16.080	0.001			
		Researches judo	0.742	0.071	12.929	0.001			
	Training programs	Provides training systematically	0.845				0.779	0.946	0.929
		Implements training programs logically	0.862	0.056	17.645	0.001			
		Teaches various tactics	0.850	0.055	17.228	0.001			
		Teaches various techniques	0.845	0.054	17.061	0.001			
		Provides training using various methods	0.848	0.054	17.160	0.001			
	Athlete understanding	Often engages in conversation to understand athletes	0.823				0.651	0.903	0.894
		Has a sound understanding of the personality and characteristics of each individual athlete	0.815	0.064	15.131	0.001			
		Pays attention to individual athletes’ hobbies and interests	0.697	0.075	12.164	0.001			
		Consults with athletes individually	0.820	0.073	15.278	0.001			
		Often praises athletes	0.811	0.067	15.016	0.001			
	Match strategy	Provides tactical instructions at appropriate times during matches	0.845				0.752	0.938	0.921
		Has a thorough grasp of opponents’ offense and defense	0.838	0.058	16.852	0.001			
		Does not miss the moments during matches when a change of strategy is needed	0.921	0.057	19.973	0.001			
		Implements bold strategies at appropriate times	0.768	0.064	14.619	0.001			
		Completely analyzes opponents	0.816	0.062	16.119	0.001			
Sport emotion	Positive emotion	I feel energized during exercise	0.846				0.763	0.941	0.936
		I feel thrilled during exercise	0.874	0.062	18.097	0.001			
		I feel happy during exercise	0.936	0.061	20.554	0.001			
		I feel emboldened during exercise	0.789	0.064	15.256	0.001			
		I feel excited during exercise	0.884	0.064	18.467	0.001			
	Negative emotion	I feel perturbed during exercise	0.774				0.649	0.902	0.910
		I feel anxious during exercise	0.890	0.077	15.256	0.001			
		I feel frustrated during exercise	0.824	0.084	13.913	0.001			
		I feel bored during exercise	0.746	0.081	12.348	0.001			
		I feel reserved during exercise	0.858	0.080	14.625	0.001			
Perceived performance	Performance success	I feel successful when I participate diligently	0.821				0.727	0.914	0.888
		I feel successful when I try my hardest	0.828	0.065	15.025	0.001			
		I feel successful when I use a new technique with good results	0.869	0.060	16.060	0.001			
		I feel successful when I think I performed well in competition	0.751	0.065	13.146	0.001			
	Performance maturation	I have ample competitive experience in judo	0.666				0.624	0.891	0.871
		I have thoroughly learned the rules of judo competition	0.747	0.087	10.361	0.001			
		I am able to solve judo-related problems myself	0.861	0.089	11.607	0.001			
		I could be provided with judo-related opportunities that I could handle myself	0.831	0.087	11.302	0.001			
		I always complete the judo-related tasks assigned to me	0.731	0.081	10.162	0.001			
	Psychological maturation	I have a strong need for achievement in judo	0.768				0.681	0.895	0.876
		I make persistent efforts until I achieve my goals	0.819	0.079	13.635	0.001			
		I think that exercise, in itself, is an enjoyable activity	0.826	0.085	13.787	0.001			
		I frequently and actively participate in team activities	0.786	0.086	12.984	0.001			

β: standardized regression weights; S.E.: standard error; C.R. (t): critical ratio; p: p-value; AVE: average variance extracted; CR: construct reliability; α: Cronbach’ α. χ^2^ = 1728.327, df = 1,035, *p* < 0.001, SRMR = 0.045, TLI = 0.928, CFI = 0.934, RMSEA = 0.052.

### 4.2 Differences in responses between athletes with less than and more than 5 years of experience

As more than half of the sample had less than 5 years of practice experience, concerns arose regarding the reliability of their judgment on the technical knowledge of judo coaches. To address this, we compared responses between athletes with less than 5 years of experience and those with more than 5 years of experience. While some differences in perception were observed, they were not significant, suggesting that even less experienced athletes were able to provide meaningful assessments of coaching knowledge.

### 4.3 Correlation analysis

We performed correlation analysis to investigate the correlations between the subfactors used in this study. The results are presented in Table 4.

**Table 4:** Correlation analysis results.

Variable	Sub-factors	*M*	*SD*	1	2	3	4	5	6	7	8	9	10
Coaching knowledge	1. Training methods	4.107	0.806										
	2. Coaching theory	4.222	0.740	0.763***									
	3. Training programs	4.169	0.760	0.793***	0.846***								
	4. Athlete understanding	3.924	0.802	0.688***	0.749***	0.781***							
	5. Match strategy	4.006	0.774	0.746***	0.816***	0.840***	0.837***						
Sport emotion	6. Positive emotion	3.490	0.877	0.505***	0.453***	0.479***	0.483***	0.490***					
	7. Negative emotion	2.308	0.905	−0.384***	−0.285***	−0.320***	−0.280***	−0.282***	−0.414***				
Perceived performance	8. Performance satisfaction	4.073	0.756	0.568***	0.583***	0.613***	0.547***	0.596***	0.595***	−0.366***			
	9. Performance maturation	3.730	0.751	0.391***	0.411***	0.396***	0.467***	0.481***	0.550***	−0.254***	0.482***		
	10. Psychological maturation	3.830	0.781	0.521***	0.510***	0.513***	0.514***	0.553***	0.746***	−0.373***	0.664***	0.686***	

M, mean; SD, standard deviation. ***p < 0.001.

First, we observed significant positive correlations between the coaching knowledge subfactors of training methods, coaching theory, training programs, athlete understanding, and match strategy, and the sport emotion subfactor of positive emotion. Meanwhile, we observed significant negative correlations between all subfactors of coaching knowledge and negative emotion (*p* < 0.001). Second, we observed significant positive correlations between all subfactors of coaching knowledge and performance success, performance maturation, and psychological maturation subfactors of perceived performance (*p* < 0.001). Third, we observed significant positive correlations between positive emotion and all subfactors of perceived performance and significant negative correlations between negative emotion and all subfactors of perceived performance (*p* < 0.001).

While discussing multicollinearity in correlation analysis, Kline (2011) advised that correlation coefficients should not exceed 0.850. Upon applying this criterion to our results, we observed correlation coefficients of −0.254–0.846, which did not exceed the criterion, demonstrating that there was no problem of multicollinearity.

### 4.4 Testing the fit of the research model

We used structural equation modeling to test our hypotheses. When testing the fit of a structural equation model, previous studies (Bae, 2017; Bentler, 1990; Browne and Cudeck, 1992; Kim et al., 2015) highlighted the importance of considering not only the sensitivity to sample size and the simplicity of the model but also the need for clear criteria for interpretation. Thus, we used the SRMR (≤0.08), TLI (≥0.9), CFI (≥0.9), and RMSEA (≤0.1) criteria proposed in these studies to test the fit of our research model. We observed fit indices of *χ*^2^(*df*) = 258.255(129)/*p* < 0.001, SRMR = 0.053, TLI = 0.961, CFI = 0.967, and RMSEA = 0.064, which satisfied the fit criteria, confirming that our research model had a good fit.

### 4.5 Hypothesis testing

**Figure 2:**
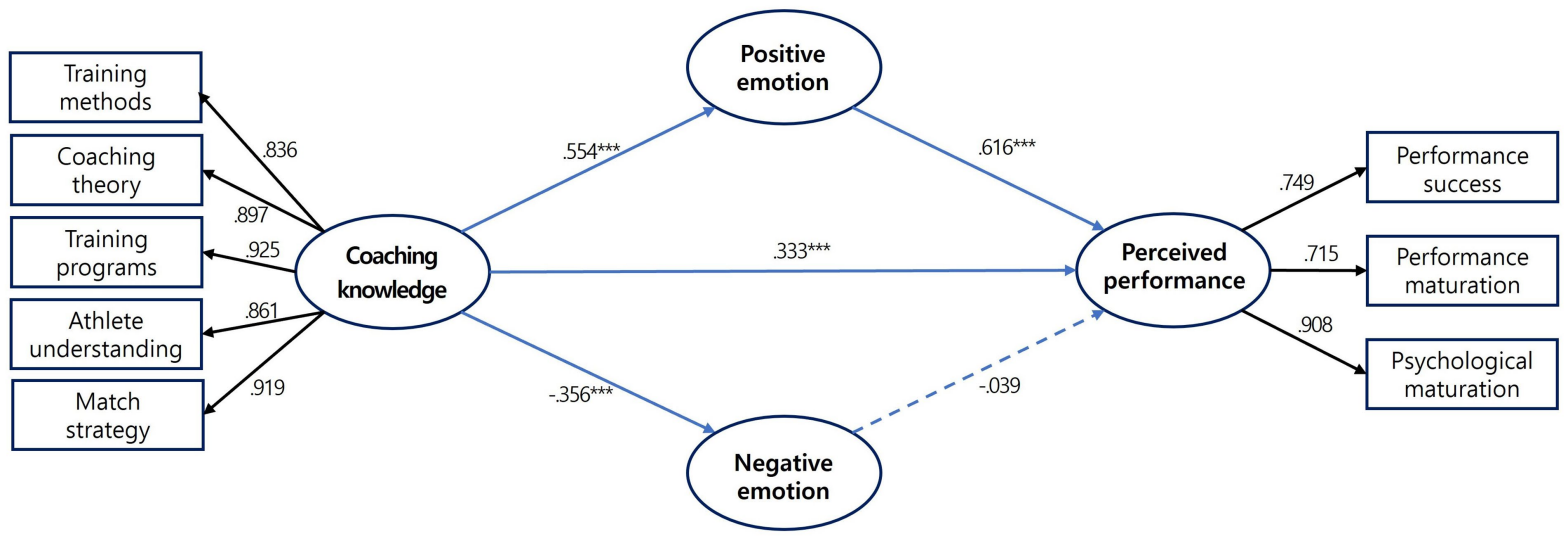
Path diagram of the structural equation modeling results. ***p < 0.001.

This study aimed to investigate the structural relationships between the coaching knowledge of judo coaches and the sport emotion and perceived performance of judoka. Table 5 and Figure 2 present the results of hypothesis testing in alignment with the study objectives.

*H1.* The coaching knowledge of judo coaches influences the sport emotion of athletes.

*H1-1*. When we analyzed the relationship between coaching knowledge and positive emotion, the path coefficient was 0.554 (*t* = 8.903), and the coaching knowledge of judo coaches significantly increased athletes’ positive emotion (*p* < 0.001, 95% CI [0.450, 0.638]).

*H1-2.* When we analyzed the relationship between coaching knowledge and negative emotion, the path coefficient was −0.356 (*t* = −5.412), and the coaching knowledge of judo coaches significantly decreased athletes’ negative emotion (*p* < 0.001, 95% CI [−0.505, −0.205]).

**Table 5:** Path analysis and fit index of the research model.

H	Path	*β*	S. E.	C. R. (*t*)	*sig*	95% CI	Result	Direct effect	Indirect effect	Total effect
H1-1	Coaching knowledge → positive emotion	0.554	0.067	8.903	0.001	[0.450, 0.638]	Accept	0.554	-	0.554
H1-2	Coaching knowledge → negative emotion	−0.356	0.085	−5.412	0.001	[−0.505, −0.205]	Accept	−0.356	-	−0.356
H2	Coaching knowledge → perceived performance	0.333	0.045	5.950	0.001	[0.563, 0.792]	Accept	0.318	0.369	0.687
H3-1	Positive emotion → perceived performance	0.638	0.051	8.909	0.001	[0.495, 0.723]	Accept	0.638	-	0.638
H3-2	Negative emotion → perceived performance	−0.029	0.030	−0.794	0.427	[−0.163, 0.078]	Reject	−0.029	-	−0.029

β: standardized path coefficient; S.E: standard error; C.R. (t): critical ratio; 95% CI: 95% confidence interval. Fit Index: χ^2^ = 258.255, df = 129, *p* < 0.001, SRMR = 0.053, TLI = 0.961, CFI = 0.967, and RMSEA = 0.064.

Thus, the first hypothesis of our study was supported.

*H2.* The coaching knowledge of judo coaches increased athletes’ perceived performance.

*H2.* When we analyzed the relationship between coaching knowledge and perceived performance, the path coefficient was 0.333 (*t* = 5.950), and the coaching knowledge of judo coaches significantly increased athletes’ perceived performance (*p* < 0.001, 95% CI [0.563, 0.792]).

Thus, the second hypothesis of our study was supported.

*H3.* The sport emotion of judoka influences their perceived performance.

*H3-1*. When we analyzed the relationship between positive emotion and perceived performance, the path coefficient was 0.638 (*t* = 8.909), and the positive emotion of judoka significantly increased their perceived performance (*p* < 0.001, 95% CI [0.495, 0.723]).

*H3-2*. When we analyzed the relationship between negative emotion and perceived performance, the path coefficient was −0.029 (*t* = −0.794), and the negative emotion of judoka did not decrease their perceived performance.

Thus, the third hypothesis of our study was partially supported.

## 5 Discussion

This study aimed to investigate the relationships between the coaching knowledge of judo coaches and the sport emotion and perceived performance of judoka. First, the coaching knowledge of judo coaches influenced the sport emotion of judoka. Coaches leverage expert knowledge to optimize both training and competitive outcomes. This underscores the relationship between expert coaching knowledge and athletes’ emotional states. The findings uniquely demonstrate that coaching knowledge affects perceived performance through the mediating role of sport emotion. This mechanism had not been tested in earlier studies. Based on these prior studies, we initially investigated the relationship between judo coaches’ coaching knowledge and athletes’ sport emotion and found that coaching knowledge increased athletes’ positive emotion and decreased athletes’ negative emotion. These observed mechanisms—systematic training design, emotional support, and strategic feedback—though studied here within Korean judo, likely underpin effective coaching practices in other sports and cultural settings as well.

These findings are difficult to discuss in depth because we were unable to find other studies investigating the relationship between the coaching knowledge of judo coaches and the sport emotion of athletes. However, our findings are consistent with a report by Nam and Cho (2014) that judo coaches’ coaching knowledge positively influences immersion, inducing internal changes in athletes that lead them to trust their coaches and to focus and participate actively during training. Our results align with a study by Nam et al. (2023), who reported that coaching knowledge positively influences exercise passion, which is a motivating force that drives athletes to achieve their personal goals. Additionally, a study by Kim and Choi (2017), reported that the perception of a judo coach as possessing good coaching knowledge could increase exercise behaviors, including responding to situational demands to achieve one’s goals, expressive interest and proactivity towards one’s exercise, and continually participating in exercise.

Coaching knowledge, which can be considered the expertise of sports coaches and includes training methods, coaching theory, and match strategy, is not merely the act of conveying and teaching movements or knowledge to athletes but can also be understood as the complex interaction of diverse factors, including the coach’s attitude, beliefs, and personality (Jeong and Yoon, 2023). In other words, one interpretation of our results is that when athletes believe in and positively accept this coaching knowledge, it creates a positive synergy with their emotions, including their feelings about exercise, their mood, posture, and belief. In contrast, distrust in the coach’s coaching knowledge can lead to the formation of negative emotions, such as psychological frustration, anxiety, boredom, and a passive attitude. Thus, judo coaches need effective, expert coaching knowledge, which is an essential element associated with various internal factors for athletes, including their sport emotion.

Second, the coaching knowledge of judo coaches increased athletes’ perceived performance. This finding is consistent with Cha and Jo (2017) and Nam et al. (2023) who have demonstrated that expert coaching practices enhance athletes’ self-evaluations of success and development. It highlights how specialized coaching knowledge drives effective training and performance appraisal.

Athletic performance is not achieved solely through the athlete’s personal efforts but is the product of innumerable situational variables, and coaching-related variables are highly closely related to athletic performance (Yang and Nam, 2019). Thus, the importance of coaches in sports is being increasingly recognized, producing not only a quantitative increase in research on coaches but also outcomes demonstrating that coaches can directly help improve athletes’ athletic performance (Álvarez et al., 2009; Chung and Huh, 2006; Deakin and Cobley, 2003; Horn et al., 2001; Song, 2016; Vallée and Bloom, 2005). As depicted in our study, high-quality coaching knowledge of coaches is associated with high-quality performance in athletes. Thus, similar to the adage that the quality of education is only as good as the teacher, for judoka to achieve a high level of perceived performance, it is essential for judo coaches to persistently strive to expand their coaching knowledge. Notably, such efforts by coaches are likely to influence not only improvements in athletes’ performance outcomes but also their broader development, including academic or career progression. This is supported by Nam et al. (2023), who found that systematic coaching efforts enhance not only athletes’ technical and psychological competencies but also their motivation, self-regulation, and long-term commitment, which are transferable to educational and professional settings.

Third, only positive sport emotion increased perceived performance, aligning with Park and Chun (2018). This reflects judo’s specific emotional dynamics, where experiencing positive emotions strongly enhances athletes’ self-appraisal. This suggests that sport emotion may play a mediating role in the relationship between coaching knowledge and perceived performance.

Our results were similar to those of a study by Lee (2012), which reported that the positive emotion of pride had positive effects on perceived performance, and that the negative emotion of sadness had negative effects on perceived performance. Although our study did not find a significant effect of negative emotion overall, this contrast may indicate that the impact of specific emotions—such as sadness—can vary by sport or context.

In summary, when athletes experience more positive emotions, they rate their own perceived performance more positively, demonstrate increased confidence in their capabilities, and psychologically exhibit a stronger desire to achieve difficult targets in sports, leading to active participation not only in personal training but also in team activities. However, our study found no significant relationship between negative emotion and perceived performance among judoka. This absence of a predictive effect may reflect both cultural norms in Korean judo that favor emotional restraint and differences in experience level: seasoned judoka often reappraise negative emotions as useful feedback, while less experienced athletes may feel hindered, resulting in a net null effect (Guassi Moreira et al., 2022; Roos and Bennett, 2023; Yeh et al., 2020). This result may indicate that the negative emotions experienced by these athletes are effectively managed or counterbalanced by adaptive coping strategies inherent in their training environment.

In other words, the lack of a significant negative impact might reflect the athletes’ ability to regulate adverse emotions, thereby minimizing their potential disruptive effects on performance. This suggests that the impact of negative emotion on performance may differ depending on sport-specific characteristics. For example, taekwondo, as a striking-based martial art, requires rapid decision-making and continuous offensive action, which may make athletes more vulnerable to the disruptive effects of negative emotions (Park and Chun, 2018). In contrast, judo is a grappling-based sport that emphasizes body control, balance, and the exploitation of opponents’ movements through strategic maneuvers (Nam et al., 2024). Unlike striking sports, which demand immediate reactions and aggression, judo often requires sustained physical contact, patience, and the ability to anticipate and counter opponents’ techniques (Kons et al., 2018; Mazzanti et al., 2025). These demands foster a performance environment where emotional regulation and composure are integral to success (Lane et al., 2012; Robazza et al., 2023). As a result, judoka may be trained, both physically and psychologically, to reinterpret or suppress negative emotions in order to maintain clarity and tactical focus. This distinction in performance demands and training philosophies may help explain why negative emotions are less disruptive to performance in judo compared to other combat sports.

Korean judoka, in particular, may have developed coping mechanisms that minimize the adverse effects of negative emotions on performance due to their intensive training culture. Future research should further investigate these differences by comparing the role of negative emotion in various combat sports. For instance, qualitative interviews with elite judoka could provide deeper insights into how they perceive and manage negative emotions during competition. Additionally, physiological measures such as cortisol or heart rate variability could be used to objectively assess stress responses and their correlation with performance outcomes.

Future research should investigate whether these cultural and sport-specific factors uniquely shape this finding, particularly in comparison to athletes from different regions or sporting disciplines. Moreover, future studies could incorporate objective physiological measures during competition, such as heart rate variability, cortisol levels, or other stress biomarkers, to complement self-reported data. These measures would provide a more comprehensive understanding of the athletes’ physiological responses to negative emotions and their potential impact on performance.

So and Ha (2020) reported that athletes need to control and regulate themselves mentally and physically and that the ability to control sport emotion results in successful performance in practice and optimal performance in competition. Therefore, judoka should attempt to maintain positive emotions as far as possible by controlling or regulating their own emotions, and this can ultimately form the foundation for outstanding athletic performance. In other words, increasing positive emotions for athletes may lead to heightened feelings of exhilaration, energy, happiness, excitement, and confidence during training, which consequently increases the success and maturation of athletic performance, as well as psychological maturation. To ensure strong perceived performance, judoka should regulate their negative emotions and maintain a high level of positive emotion. Hence, judo coaches need to acquire high-quality coaching knowledge, and athletes need to be receptive to this coaching knowledge. This is because high-quality coaching knowledge from judo coaches not only enhances athletes’ positive emotions and reduces negative emotions but also serves as a major factor, alongside positive emotion, in fostering strong perceived performance.

Furthermore, while this study examined the structural relationships among coaching knowledge, sport emotion, and perceived performance, future research should include more quantitative performance measures. For example, detailed analysis of performance success, performance maturation, and psychological maturation could reveal differential impacts of coaching practices and athlete emotional responses on various dimensions of athletic performance. In our study, although these sub-dimensions were measured, a more granular analysis may uncover specific patterns—such as whether performance success is more strongly influenced by immediate emotional responses compared to long-term performance maturation. Moreover, integrating these quantitative variables with objective performance data (e.g., competition results, training outcomes) may help in triangulating the findings and enhancing the validity of the conclusions drawn. This integrated approach could facilitate a deeper understanding of the complex interplay between coaching, emotion, and performance, providing clearer guidance for both practitioners and future research.

### 5.1 Limitations

Despite the strengths of this study, some limitations must be acknowledged. First, due to logistical constraints, the questionnaire was administered at a single time point (within 24 h after the competition). This single time-point measurement limits our ability to capture both anticipatory and reflective evaluations, as athletes’ perceptions of their performance might evolve over time and immediate post-competition responses could differ from those given after a period of reflection. Future studies should consider multi-stage assessments for a more comprehensive analysis.

Second, this study did not conduct gender-based subgroup analyses, not only to maintain focus on the structural relationships among coaching knowledge, sport emotion, and perceived performance, but also because of the considerable imbalance in the overall gender ratio. However, future studies may benefit from examining whether these relationships differ by gender, especially considering the potential psychological and emotional differences between male and female athletes.

Third, although analyses validated the responses from less experienced athletes, the impact of their limited exposure to coaching remains a concern. Furthermore, the strong interpersonal and affective bond between coaches and athletes may have led to a social desirability bias, potentially inflating positive assessments of coaching knowledge and perceived performance. Further research should consider alternative assessment methods, such as independent evaluations from experienced coaches.

Finally, considering the natural emotional bond between athletes and their coaches, athletes’ subjective perceptions may have been influenced by their personal relationship with their coach.

Although team and competition levels were collected as background information, they were not included as covariates in the structural equation model due to several methodological considerations. First, the substantial imbalance across team levels (e.g., middle school, high school, university, business) could bias results and compromise parameter stability. Second, adding these categorical variables alongside core psychological constructs—coaching knowledge, sport emotion, and perceived performance—risked multicollinearity, potentially distorting structural interpretations. Third, the study’s primary focus was on general psychological mechanisms, not subgroup differences; including these variables would have shifted the analytical emphasis. We therefore acknowledge this exclusion as a limitation.

Future research should consider including team and competition levels as covariates or moderators when more balanced samples are available, to examine potential subgroup effects more rigorously. Additionally, to reduce response biases—such as social desirability or limited evaluative experience—researchers may incorporate third-party assessments (e.g., from independent coaches or experts) or adopt longitudinal study designs that capture how athletes’ perceptions evolve over time.

### 5.2 Practical implications

These findings offer several practical insights for coaches, athletes, and training program developers. First, the significant role of coaching knowledge suggests that ongoing coach education should emphasize not only technical instruction but also communication skills, emotional intelligence, and athlete-centered pedagogy. Well-structured continuing education programs can help coaches better support athletes’ emotional states and perceived performance (Camiré et al., 2018; Paquette and Trudel, 2018; Zajonz et al., 2024).

Second, the finding that positive emotion enhances perceived performance highlights the value of emotional regulation training for athletes. Integrating mental skills training—such as emotional awareness, cognitive reappraisal, and relaxation techniques—into daily practice may help judoka maintain optimal emotional states during competition (Giles et al., 2018; Robazza et al., 2023).

Finally, given the null effect of negative emotion, programs may benefit from identifying how experienced judoka reinterpret or neutralize negative feelings. Peer mentoring and reflective practice sessions could help less experienced athletes develop similar coping strategies.

## 6 Conclusion

This study examined the structural relationships between judo coaches’ coaching knowledge and judoka’s sport emotion and perceived performance. In this sample of 249 South Korean judoka, higher levels of coaching knowledge significantly increased positive emotion, decreased negative emotion, and enhanced perceived performance. In addition, only positive emotion significantly enhanced perceived performance, while negative emotion did not influence it. These findings indicate that coaching knowledge and positive sport emotion are key psychological contributors to perceived performance in competitive sports settings.

Importantly, our findings suggest these associations are not confined to Korean judo but represent core coaching mechanisms that likely apply to sports coaches more broadly, whether in team, individual, or combat disciplines globally. Furthermore, while the specific context of Korean judo coaching—characterized by disciplined hierarchical structures and integrated technical-emotional guidance—highlights distinctive cultural nuances, it also underscores universal coaching principles (e.g., knowledge transfer, emotional regulation, strategic feedback) that warrant investigation across diverse sporting and national contexts.

Because this study focused on a single cultural and sport-specific context, the generalizability of its findings should be interpreted with caution. Further research could explore whether similar patterns emerge in other sports or national settings. Additionally, future studies could expand the model to include other coach- and athlete-related psychological variables, and adopt mixed-methods approaches to explore the dynamic interplay between coaching knowledge, emotional processes, and athlete performance over time.

## Data Availability

The datasets generated for this study are available from the corresponding author upon reasonable request.

## References

[ref1] Álvarez M. S. Balaguer I. Castillo I. Duda J. L. (2009). Coach autonomy support and quality of sport engagement in young soccer players. Span. J. Psychol. 12, 138–148. doi: 10.1017/s1138741600001554, PMID: 19476227

[ref2] Andrzejewski M. Konefał M. Chmura P. Kowalczuk E. Chmura J. (2016). Match outcome and distances covered at various speeds in match play by elite German soccer players. Int. J. Perform. Anal. Sport 16, 817–828. doi: 10.1080/24748668.2016.11868930

[ref3] Bae B. R. (2017). Structural equation modeling with Amos 24: principles and practice. Seoul, Korea: Chung-Ram Press.

[ref4] Bentler P. M. (1990). Comparative fit indexes in structural models. Psychol. Bull. 107, 238–246. doi: 10.1037/0033-2909.107.2.238, PMID: 2320703

[ref5] Bois J. E. Lalanne J. Delforge C. (2009). The influence of parenting practices and parental presence on children’s and adolescents’ pre-competitive anxiety. J. Sports Sci. 27, 995–1005. doi: 10.1080/02640410903062001, PMID: 19847683

[ref6] Browne M. W. Cudeck R. (1992). Alternative ways of assessing model fit. Sociol. Methods Res. 21, 230–258. doi: 10.1177/0049124192021002005

[ref7] Camiré M. Rathwell S. Felber Charbonneau E. Kendellen K. (2018). Evaluation of the pilot implementation of the coaching for life skills program. Int. Sport Coaching J. 5, 227–236. doi: 10.1123/iscj.2018-0006

[ref8] Cha J. K. Jo W. S. (2017). Physical education high school student athletes’ perception of coaching knowledge, leadership and organizational exercise structure. J. Coach. Dev. 19, 3–13.

[ref9] Cho B. C. (2023). Structural relationships among mindset, grit, exercise flow, and performance in junior golfers (Dissertation/Ph.D.). Yongin University.

[ref10] Choi K. Y. Ha J. H. (2020). The effects of golf coach’s coaching knowledge on trust of coach and exercise volitions of players. Korean J. Sports Sci. 29, 445–455. doi: 10.35159/kjss.2020.02.29.1.445

[ref11] Choi H. Jeong Y. Kim S.-K. (2020). The relationship between coaching behavior and athlete burnout: mediating effects of communication and the coach–athlete relationship. Int. J. Environ. Res. Public Health 17:8618. doi: 10.3390/ijerph17228618, PMID: 33233544 PMC7699703

[ref12] Choi M. S. Yoo J. I. (2011). The causal relationship among emotional intelligence, team cohesion, and athlete satisfaction of team sports players. J. Coach. Dev. 13, 13–22.

[ref13] Chung J. H. (2004). A qualitative study on coaching knowledge of successful basketball coaches in woman’s high schools. Korean J. Sport Sci. 15, 94–108.

[ref14] Chung I. M. (2017). Mentoring affects college golfers’ passion and psychology of their profession, and performance in their recognition. Korean J. Sports Sci. 26, 509–521. doi: 10.35159/kjss.2017.12.26.6.509

[ref15] Chung J. H. Huh J. H. (2006). Structural validity of coaching knowledge questionnaire. Korean J. Sport Psychol. 17, 57–69.

[ref16] Coswig V. S. Gentil P. Bueno J. C. A. Follmer B. Marques V. A. Del Vecchio F. B. (2018). Physical fitness predicts technical-tactical and time-motion profile in simulated judo and Brazilian Jiu-Jitsu matches. PeerJ 6:e4851. doi: 10.7717/peerj.4851, PMID: 29844991 PMC5971839

[ref17] Crombie D. Lombard C. Noakes T. (2009). Emotional intelligence scores predict team sports performance in a national cricket competition. Int. J. Sports Sci. Coach. 4, 209–224. doi: 10.1260/174795409788549544

[ref18] De Jesus K. Fernandes R. J. Vilas-Boas J. P. Sanders R. Ribeiro J. Figueiredo P.. (2016). The effect of intensity on 3-dimensional kinematics and coordination in front-crawl swimming. Int. J. Sports Physiol. Perform. 11, 768–775. doi: 10.1123/ijspp.2015-0465 26658832

[ref19] Deakin J. M. Cobley S. (2003). “An examination of the practice environments in figure skating and volleyball: a search for deliberate practice” in Expert performance in sports: Advances in research on sport expertise. eds. Starkes J. Ericsson K. A. (Champaign, IL, USA: Human Kinetics Publishers), 115–135.

[ref20] Duda J. L. (1989). Relationship between task and ego orientation and the perceived purpose of sport among high school athletes. J. Sport Exerc. Psychol. 11, 318–335. doi: 10.1123/jsep.11.3.318

[ref21] Ericsson K. A. Smith J. (1991). Toward a general theory of expertise: Prospects and limits. Cambridge, England: Cambridge University Press.

[ref22] Franchini E. Artioli G. G. Brito C. J. (2013). Judo combat: time-motion analysis and physiology. Int. J. Perform. Anal. Sport 13, 624–641. doi: 10.1080/24748668.2013.11868676

[ref23] Fransen K. Boen F. Vansteenkiste M. Mertens N. Vande Broek G. (2018). The power of competence support: the impact of coaches and athlete leaders on intrinsic motivation and performance. Scand. J. Med. Sci. Sports 28, 725–745. doi: 10.1111/sms.12950, PMID: 28730741

[ref24] Gellatly I. R. Meyer J. P. (1992). The effects of goal difficulty on physiological arousal, cognition, and task performance. J. Appl. Psychol. 77, 694–704. doi: 10.1037/0021-9010.77.5.694, PMID: 1429347

[ref25] Giles G. E. Eddy M. D. Cantelon J. A. Mahoney C. R. Kanarek R. B. Urry H. L.. (2018). Cognitive reappraisal reduces perceived exertion during endurance exercise. Motiv. Emot. 42, 482–496. doi: 10.1007/s11031-018-9697-z

[ref26] Ginevičienė V. Utkus A. Pranckevičienė E. Semenova E. A. Hall E. C. R. Ahmetov I. I. (2022). Perspectives in sports genomics. Biomedicine 10:298. doi: 10.3390/biomedicines10020298, PMID: 35203507 PMC8869752

[ref27] Guassi Moreira J. F. Ninova E. Silvers J. A. Parkinson C. Sahi R. (2022). Performance and belief-based emotion regulation capacity and tendency: mapping links with cognitive flexibility and perceived stress. Emotion 22, 653–668. doi: 10.1037/emo0000768, PMID: 32463278

[ref28] Guth L. M. Roth S. M. (2013). Genetic influence on athletic performance. Curr. Opin. Pediatr. 25, 653–658. doi: 10.1097/MOP.0b013e3283659087, PMID: 24240283 PMC3993978

[ref29] Ha J. Hong Y. (2022). The differences of elite tennis players’ exercise emotion and sport burnout according to cluster types based on the perfectionism orientation. Korea J. Sport 20, 813–826. doi: 10.46669/kss.2022.20.2.069

[ref30] Hair J. F. Black B. Babin B. J. Anderson R. Tatham R. L. (2006). Multivariate data analysis: United States edition. 6th Edn. New Jersey, USA: Pearson.

[ref31] Hanin Y. L. (2000). Emotions in sport. Champaign: Illinois Human kinetics.

[ref32] Hanin Y. L. (2004). “Emotion in sport: an individualized approach” in Encyclopedia of applied psychology. ed. Spielberger C. D., vol. 1 (Amsterdam, Netherlands: Elsevier Academic Press), 739–750.

[ref33] Hong Y. J. Choi Y. R. (2022). Mediating effect of athletic emotions on the relationship between leader’s autonomy support and exercise exhaustion in badminton players. Korea J. Sport 20, 805–817. doi: 10.46669/kss.2022.20.3.070

[ref34] Hong D. S. Noh G. T. Kim S. H. (2013). Based on possible associations of tennis coach’s mentoring function with players’ perceived sense of freedom, their self-realization and exercise emotion. J. Korean Wellness Soc. 8, 245–269.

[ref35] Horn T. S. Lox C. L. Labrador F. (2001). “The self-fulfilling prophecy theory: when coaches’ expectations become reality” in Applied sport psychology: Personal growth to peak performance. ed. Williams J. (Mountain View, CA: Mayfield), 68–81.

[ref36] Hwang G. Y. (2018). Effects of achievement goal orientation on exercise emotion and exercise adherence among university students. Korean J. Sport Sci. 16, 1–14.

[ref37] Jackson S. A. Thomas P. R. Marsh H. W. Smethurst C. J. (2001). Relationships between flow, self-concept, psychological skills, and performance. J. Appl. Sport Psychol. 13, 129–153. doi: 10.1080/104132001753149865

[ref38] Jang J. H. Kong S. B. (2022). The relationship between university taekwondo players’ tenacity, exercise passion, and perceived performance. J. Coach. Dev. 24, 178–185. doi: 10.47684/jcd.2022.09.24.4.178

[ref39] Jeong H. Yoon S. J. (2023). Police use of force and the relationship among instructor’s professionalism, learning flow, and educational satisfaction. J. Korean Dance Soc. 25, 89–103. doi: 10.35277/kama.2023.25.3.89

[ref40] Kim S. I. (2011). The relationship among the athletes coach’s leadership types, sports emotion and collective efficacy of team athletes players. Korean J. Sport Sci. 9, 13–26.

[ref41] Kim B. (2012). The relationship among emotional leadership of elite athlete coach, athletes’ emotions, team climate and team effectiveness. Korean J. Sport Sci. 21, 75–89.

[ref42] Kim Y. Bang E. (2022). The effect of badminton players perceived performance on self-esteem and resilience. ISS 40, 145–153. doi: 10.46394/ISS.40.1.14

[ref43] Kim S.-G. Choi M.-S. (2014). An exploration of the causal relationship among transactional leadership, coaches’ emotional intelligence, and athlete satisfaction in soccer teams. J. Korea Contents Assoc. 14, 450–462. doi: 10.5392/JKCA.2014.14.09.450

[ref44] Kim W. S. Choi Y. J. (2017). The relationship among coaching knowledge, trust of coach, and sport behaviors of judo athletes. Korean J. Sport Sci. 1, 1–11.

[ref45] Kim D. H. Choi S. O. Kim B. J. (2011). Verification of a causal model among emotion origination, emotion regulation and sports performance. J. Sport Leis. Stud. 43, 865–880. doi: 10.51979/KSSLS.2011.02.43.865

[ref46] Kim J. Y. Jang M. H. Park S. R. (2023). Examining the role of coach trust between authentic leadership and sport psychology in student athletes. J. Sport Leis. Stud. 91, 109–119. doi: 10.51979/KSSLS.2023.01.91.109

[ref47] Kim J. H. Kim M. K. Hong S. H. (2015). A writing the structural equation model analysis. Republic of Korea: Communication Books.

[ref48] Kim S. D. Lee Y. K. (2012). The effects of college athletes’ emotional intelligence on athlete satisfaction and athletic performance. Korean J. Sport Sci. 10, 37–48.

[ref49] Kim D. E. Nam K. W. (2020). The relationship between instructing characteristics and athletic performance of gymnasts: focus on mediating effect of self-management. Korea J. Sport 18, 1039–1048. doi: 10.46669/kss.2020.18.2.091

[ref50] Kim Y. J. Tak M. (2025). Coaching transitions across borders: the pursuit of individuals advancing coaching careers in the competitive global landscape of Olympic sports. Int. Sport Coach. J. 12, 116–125. doi: 10.1123/iscj.2023-0058

[ref51] Kline R. B. (2011). Principles and practice of structural equation modeling. 3rd Edn. New York: Guilford Press.

[ref52] Kons R. L. Detanico D. Dal Pupo J. Ache-Dias J. (2018). Female judo athletes’ physical test performances are unrelated to technical-tactical competition skills. Percept. Mot. Skills 125, 802–816. doi: 10.1177/0031512518777586, PMID: 29788859

[ref53] Lane A. M. Beedie C. J. Jones M. V. Uphill M. Devonport T. J. (2012). The BASES expert statement on emotion regulation in sport. J. Sports Sci. 30, 1189–1195. doi: 10.1080/02640414.2012.693621, PMID: 22709410

[ref54] Lazarus R. S. (2000). How emotions influence performance in competitive sports. Sport Psychol. 14, 229–252. doi: 10.1123/tsp.14.3.229, PMID: 40574732

[ref55] Lazarus R. S. (2006). Emotions and interpersonal relationships: toward a person-centered conceptualization of emotions and coping. J. Pers. 74, 9–46. doi: 10.1111/j.1467-6494.2005.00368.x, PMID: 16451225

[ref56] Lee M. J. (2000). The relationship between social facilitation effect and satisfaction on the cycle racing performance along with the cycle racing player class. Korea National University (Master’s thesis).

[ref57] Lee Y. M. (2012). Effect of the self-management strategies on passionate movement, exercise affect, and perceived performance in college taekwondo players. Dankook University (Master’s thesis).

[ref58] Lee S. J. Ko E. J. Kim S. S. (2019). The effect of organization-based self-esteem on student athlete’s perceived performance in sports team: focused on the affective commitment and athletic satisfaction. JKSSPE 24, 117–131. doi: 10.15831/JKSSPE.2019.24.2.117

[ref59] Lee U. K. Yoo H. S. (2023). Effect of psychological skill training on mood states, sports performance strategy, sports coping skills, and sports confidence in cyclist. J. Coach. Dev. 25, 178–186. doi: 10.47684/jcd.2023.01.25.1.178

[ref60] Lemyre P. N. Treasure D. C. Roberts G. C. (2006). Influence of variability in motivation and affect on elite athlete burnout susceptibility. J. Sport Exerc. Psychol. 28, 32–48. doi: 10.1123/jsep.28.1.32

[ref61] Mamassis G. Doganis G. (2004). The effects of a mental training program on juniors pre-competitive anxiety, self-confidence, and tennis performance. J. Appl. Sport Psychol. 16, 118–137. doi: 10.1080/10413200490437903

[ref62] Mazzanti M. Masini A. Sanmarchi F. Dallolio L. Mascherini G. (2025). Aggression and sport: a cross-sectional study on behavioral tendencies of athletes. J. Bodyw. Mov. Ther. 42, 982–988. doi: 10.1016/j.jbmt.2025.03.001 40325782

[ref63] Mccarthy P. J. (2011). Positive emotion in sport performance: current status and future directions. Int. Rev. Sport Exerc. Psychol. 4, 50–69. doi: 10.1080/1750984X.2011.560955

[ref64] Moen F. Hrozanova M. Stiles T. C. Stenseng F. (2019). Burnout and perceived performance among junior athletes-associations with affective and cognitive components of stress. Sports 7:171. doi: 10.3390/sports7070171, PMID: 31336729 PMC6680912

[ref65] Nam K. W. Cho O. S. (2014). The impact of judo student athletes’ perception of coaches’ coaching knowledge on trust of coach and sport commitment. J. Korean Soc. Phys. Educ. 53, 143–154.

[ref66] Nam K. Kim C. Yoon S. (2024). Relationship between judo coaches’ authentic leadership and judo athletes’ perceived performance: mediating effect of self-management. Int. J. Sports Sci. Coach. 19, 19–32. doi: 10.1177/17479541231190036

[ref67] Nam K. W. Lee M. J. Yang S. H. (2023). The relationship among instructor’s coaching knowledge, exercise passion and athletic performance of judokas. J. Korean Dance Soc. 25, 111–123. doi: 10.35277/kama.2023.25.4.111

[ref69] Pan P. Li F. Han B. Yuan B. Liu T. (2023). Exploring the impact of professional soccer substitute players on physical and technical performance. BMC Sports Sci. Med. Rehabil. 15:143. doi: 10.1186/s13102-023-00752-x, PMID: 37898786 PMC10612320

[ref70] Paquette K. Trudel P. (2018). Learner-centered coach education: practical recommendations for coach development administrators. Int. Sport Coach. J. 5, 169–175. doi: 10.1123/iscj.2017-0084

[ref71] Park J. W. Chun B. K. (2018). The influence of social support on self-determination and emotion, athletic performance of taekwondo players. Korean J. Growth Dev. 26, 171–179. doi: 10.34284/KJGD.2018.05.26.2.171

[ref72] Park J. H. Jeong Y. T. (2022). Analysis of ssireum athletes sports emotions and competitive state anxiety. J. Coach. Dev. 24, 80–92. doi: 10.47684/jcd.2022.06.24.2.80

[ref73] Park C. D. Kang Y. S. (2023). The relationship between stress, self-management, exercise emotion, and crisis management of wushu athletes. Korean Soc. Leisure Sci. 14, 199–213. doi: 10.37408/kjls.2023.14.1.199

[ref74] Park M. S. Kim P. J. (2016). The relationship between coaching knowledge, self-management and sport confidence of high school taekwondo players. Korean J. Sport Sci. 25, 109–123.

[ref75] Rejeski W. J. Thompson A. Brubaker P. H. Miller H. S. (1992). Acute exercise: buffering psychosocial stress responses in women. Health Psychol. 11, 355–362. doi: 10.1037//0278-6133.11.6.355, PMID: 1286654

[ref76] Robazza C. Morano M. Bortoli L. Ruiz M. C. (2023). Athletes’ basic psychological needs and emotions: the role of cognitive reappraisal. Front. Psychol. 14:1205102. doi: 10.3389/fpsyg.2023.1205102, PMID: 37519370 PMC10374325

[ref77] Roos L. G. Bennett J. M. (2023). Reappraisal and health: how habitual reappraisal and reappraisal ability interact to protect against life stress in young adults. Emotion 23, 1360–1372. doi: 10.1037/emo0001154, PMID: 36074623

[ref68] Sakalidis K. E. Ling F. C. M. Hettinga F. J. (2023). Coaching styles and sports motivation in athletes with and without Intellectual Impairments. PLOS ONE 18:e0296164. doi: 10.1371/journal.pone.0296164 38134184 PMC10745216

[ref78] So Y. H. Ha S. W. (2020). Relationship among goal orientation, sport emotional intelligence, and perceived performance of high school athletes. J. Sport Leis. Stud. 79, 327–337. doi: 10.51979/KSSLS.2020.01.79.327

[ref79] Song D. N. (2016). A structural relationship among coach’s belief, coaching knowledge, and exercise behavior perceived by Judo athletes: Youngin University.

[ref80] Song K. (2021). The effect of college taekwondo athletes’ social exclusion experience on team immersion and performance. Korea J. Sport 19, 941–950. doi: 10.46669/kss.2021.19.4.082

[ref81] Stein G. L. Daniels J. Kimiecik J. C. Jackson S. A. (1995). Psychological antecedents of flow in recreational sport. Personal. Soc. Psychol. Bull. 21, 125–135. doi: 10.1177/0146167295212003

[ref82] Stephenson M. T. Holbert R. L. Zimmerman R. S. (2006). On the use of structural equation modeling in health communication research. Health Commun. 20, 159–167. doi: 10.1207/s15327027hc2002_7, PMID: 16965253

[ref83] Vallée C. N. Bloom G. A. (2005). Building a successful university program: key and common elements of expert coaches. J. Appl. Sport Psychol. 17, 179–196. doi: 10.1080/10413200591010021

[ref84] Watson D. Clark L. A. (1994). The PANAS-X: Manual for the positive and negative affect schedule – Expanded form. Iowa City, IA: University of Iowa, Department of Psychology.

[ref85] Williams L. J. Edwards J. R. Vandenberg R. J. (2009). 12 structural equation modeling in management research: a guide for improved analysis. Acad. Manag. Ann. 3, 543–604. doi: 10.17077/48vt-m4t2

[ref86] Yang S. H. Nam K. W. (2019). The relationship between instructor’s image and performance strategies of judoka: focus on mediating effect of training attitude. Korean J. Sport Sci. 17, 1029–1038.

[ref87] Yeh N. Opitz P. Barber S. J. Suri G. (2020). The role of reappraisal success in emotional and memory outcomes. Emotion 20, 939–950. doi: 10.1037/emo0000575, PMID: 31192660

[ref88] Yin X. Zhu R. Shi X. Cai G. Jing C. Pan Q.. (2023). The effect of rhythm training on the motor coordination abilities of 8–12-year-old freestyle swimmers. PeerJ 11:e15667. doi: 10.7717/peerj.15667, PMID: 37529213 PMC10389069

[ref89] Yoo Y. Jung K. Choi H. (2018). The relationship between the athletic performance, trust in an instructor and perception of college soccer player on soccer coache’s coaching knowledge. Korean Assoc. Learn. Center. Curric. Instr. 18, 953–968. doi: 10.22251/jlcci.2018.18.23.953

[ref90] Zajonz P. Lautenbach F. Laborde S. (2024). Emotional intelligence training for sports coaches – evaluation of a time-effective online intervention and its effects on coaching efficacy. J. Appl. Sport Psychol. 36, 722–739. doi: 10.1080/10413200.2023.2296911

